# Large Vessel Occlusion Identification Through Prehospital Administration of Stroke Scales: A County-wide Emergency Medical Services Prospective Research Protocol

**DOI:** 10.7759/cureus.5931

**Published:** 2019-10-17

**Authors:** Tej G Stead, Paul R Banerjee, Latha Ganti

**Affiliations:** 1 Emergency Medicine, Brown University, Providence, USA; 2 Emergency Medicine, University of Central Florida, Orlando, USA; 3 Emergency Medicine, Envision Physician Services, Orlando, USA

**Keywords:** stroke, ems, prehospital

## Abstract

There is yet insufficient research on prehospital stroke scales, especially for identifying large vessel occlusions and severe strokes. When multiple stroke centers are available, determining which patients should go directly to a comprehensive stroke center (CSC) is critical. Delay in care transporting to a hospital not capable of treating hemorrhagic strokes and large vessel occlusions (LVOs) can be devastating. The failure rate for tissue plasminogen activator (tPA), a clot-busting drug commonly used to treat ischemic stroke that can be administered at primary stroke centers, is up to 90% for large vessel occlusions (LVOs). However, these patients can benefit from mechanical intervention, performed only at CSCs. Hemorrhagic strokes often result from ruptured aneurysms, which can benefit from coiling and clipping, procedures also typically only available at CSCs.

In order to analyze the effectiveness of certain prehospital stroke scales, our county’s emergency medical services (EMS) system designed and implemented the LVO identification through prehospital administration of stroke scales (LIT-PASS), a prospective cohort study. Our study has three phases, each phase testing a certain combination of prehospital stroke scales. The protocol, including training for every paramedic, was started in 2015, and data collection began in 2016. In Phase 1, we tested the Los Angeles motor scale (LAMS) alone from January 2016 to November 2018. In Phase 2, we administered both the LAMS and the vision, aphasia, neglect (VAN) test from December 2018 to May 2019. Phase 3 began in June 2019 and uses the balance, eyes, face, arm, speech, terrible headache/time to call 911 (BE-FAST) test as a scale, allotting one point for each category. While the “time to call 911” aspect is not part of the scale, it is included in the name for mnemonic reasons. We chose these scales because of the symptoms they cover and due to their simplicity. Phase 1 assesses only motor symptoms, Phase 2 assesses motor and additional cortical symptoms, and Phase 3 evaluates a scale that combines both components and whose acronym is a useful mnemonic for paramedics.

Each paramedic in our county’s system was given a one-hour training session on the scales each year in Phase 1 and once prior to the beginning of Phase 2 and Phase 3. Paramedics were not allowed to respond to a stroke call unless they had completed the training. This is done to avoid bias in which patients are studied, ensuring that all stroke patients are subject to our county's stroke protocol. Data were de-identified and analyzed to evaluate the effectiveness of four things: in Phases 1 and 2, the LAMS alone; in Phase 2, the VAN test alone, as well as in combination with the LAMS; and in Phase 3, the effectiveness of the BE-FAST scale.

## Introduction

Emergency medical services (EMS) systems across the country face a dilemma: when should a suspected stroke patient be transported to a comprehensive stroke center (CSC)? On one hand, CSCs can provide mechanical thrombectomy (MT) for ischemic strokes, as well as coiling and clipping for intracerebral aneurysms. On the other hand, CSCs should not be clogged up by transporting every suspected stroke patient there, and primary stroke centers (PSCs) will have no experience administering tissue plasminogen activator (tPA) if they never receive stroke transports. An additional consideration is the distance to each type of center, both from the patient's perspective (could they perhaps get some initial treatment?) as well as from the EMS perspective (in small systems, a single ambulance being out for an extended period of time could mean other patient's transports are delayed).

Large vessel occlusions (LVOs) account for less than 20% of all ischemic strokes but are the most deadly type of ischemic stroke and only have a 10%-13% recanalization rate from intravenous (IV) tPA alone on initial angiogram [1-2]. MT is the most effective treatment method for LVOs [3]. Furthermore, subarachnoid hemorrhages, a subtype of hemorrhagic strokes, are often caused by a bleeding aneurysm, which have high success rates from coiling and clipping, a procedure typically only available at comprehensive stroke centers [4]. Thus, it is important to identify LVOs and ruptured aneurysms so they can be treated at a capable hospital with the goal of treating the right patient at the right time in the right place.

There exist many stroke scales that aim to identify LVOs, but, to date, insufficient research has been performed to identify any scale as effective in the prehospital setting [5-6]. Many studies have assessed the National Institutes of Health Stroke Scale (NIHSS) as an LVO prediction tool but rarely in the prehospital setting. It may be unrealistic to expect paramedics to perform this assessment, especially in the field where every minute matters; as we know, time is brain [7]. Given that 72% of paramedics do not have more than a high school education plus a paramedic certificate program, there could potentially exist high provider variability in implementing complex scales such as the NIHSS in the prehospital setting [8]. However, the literature lacks information about the ability of paramedics to perform NIHSS, because it is generally not used in the field. Many simple scales, some of which are derived from the NIHSS, are in use, but few have been proven to be optimal and thus there is a lack of consensus on which scale to use.

In response to the lack of sufficient prehospital research and consensus on the best prehospital stroke scale, Polk County Fire Rescue (PCFR) designed and implemented the three-phase LVO identification through prehospital administration of stroke scales (LIT-PASS) prospective study.

## Materials and methods

Polk County Fire Rescue (PCFR) is the fourth largest EMS system in the state of Florida, responding to over 85,000 patient calls a year and treating over 600 stroke patients a year while covering an area of over 2,010 square miles and serving a population of 700,000. The PCFR system, in partnership with its three main municipalities, employs 800 paramedics and EMTs and transports to three CSCs and four primary stroke centers (PSCs).

**Figure 1:**
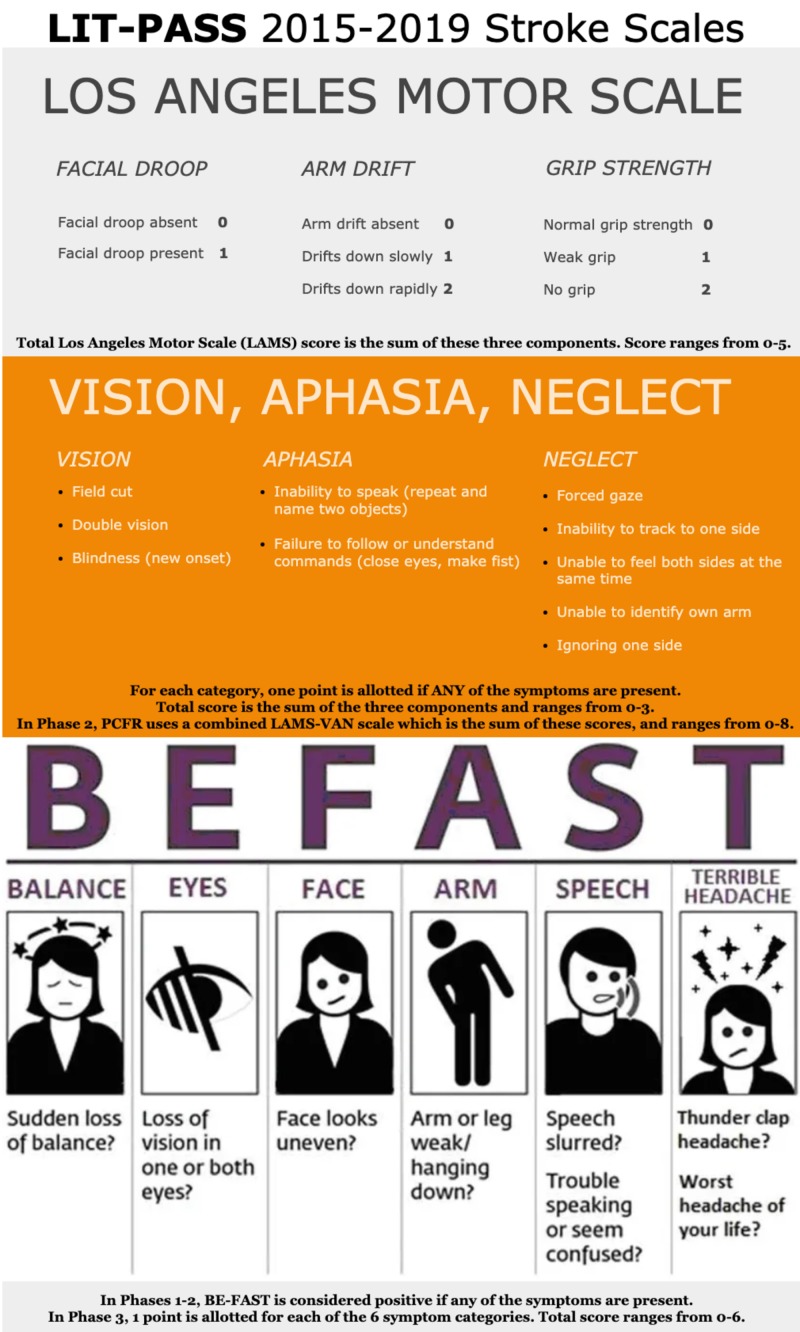
Stroke scales used by PCFR in our study. BE-FAST image sourced from [9], PCFR = Polk County Fire Rescue, BE-FAST = balance, eyes, face, arm, speech, terrible headache/time to call 911

LIT-PASS is composed of three phases. In Phase 1, we tested the Los Angeles motor scale (LAMS) alone from January 2016-November 2018. In Phase 2, we administered both the LAMS and the vision, aphasia, neglect (VAN) test from December 2018 to May 2019. Phase 3 began in June 2019 and uses the balance, eyes, face, arm, speech, terrible headache/time to call 911 (BE-FAST) test as a scale, allotting one point for each category (Figure 1). These phases were chosen because each of the three scales tested is very simple and leads to low provider variability. Phase 1 focuses exclusively on motor symptoms, Phase 2 analyzed both motor and additional cortical symptoms (in our analyses, we looked at the VAN alone, as well as in combination with the LAMS), and Phase 3 uses a combined scale that covers a broader spectrum of stroke signs and has a useful mnemonic.

**Figure 2:**
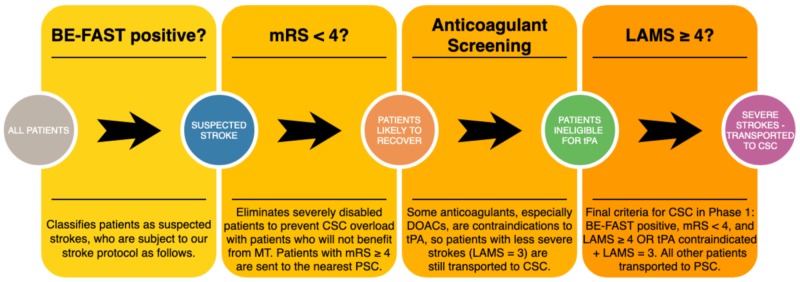
Phase 1 stroke protocol DOACs = direct oral anticoagulants, BE-FAST = balance, eyes, face, arm, speech, terrible headache/time to call 911, mRS = modified Rankin score, LAMS = Los Angeles motor scale, CSC = comprehensive stroke center, MT = mechanical thrombectomy, PSC = primary stroke center, tPA = tissue plasminogen activator

**Figure 3:**
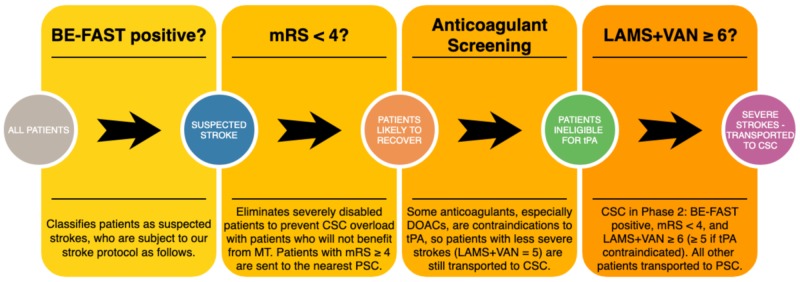
Phase 2 stroke protocol DOACs = direct oral anticoagulants, BE-FAST = balance, eyes, face, arm, speech, terrible headache/time to call 911, mRS = modified Rankin score, LAMS = Los Angeles motor scale, CSC = comprehensive stroke center, MT = mechanical thrombectomy, PSC = primary stroke center, tPA = tissue plasminogen activator, VAN = vision, aphasia, neglect

**Figure 4:**
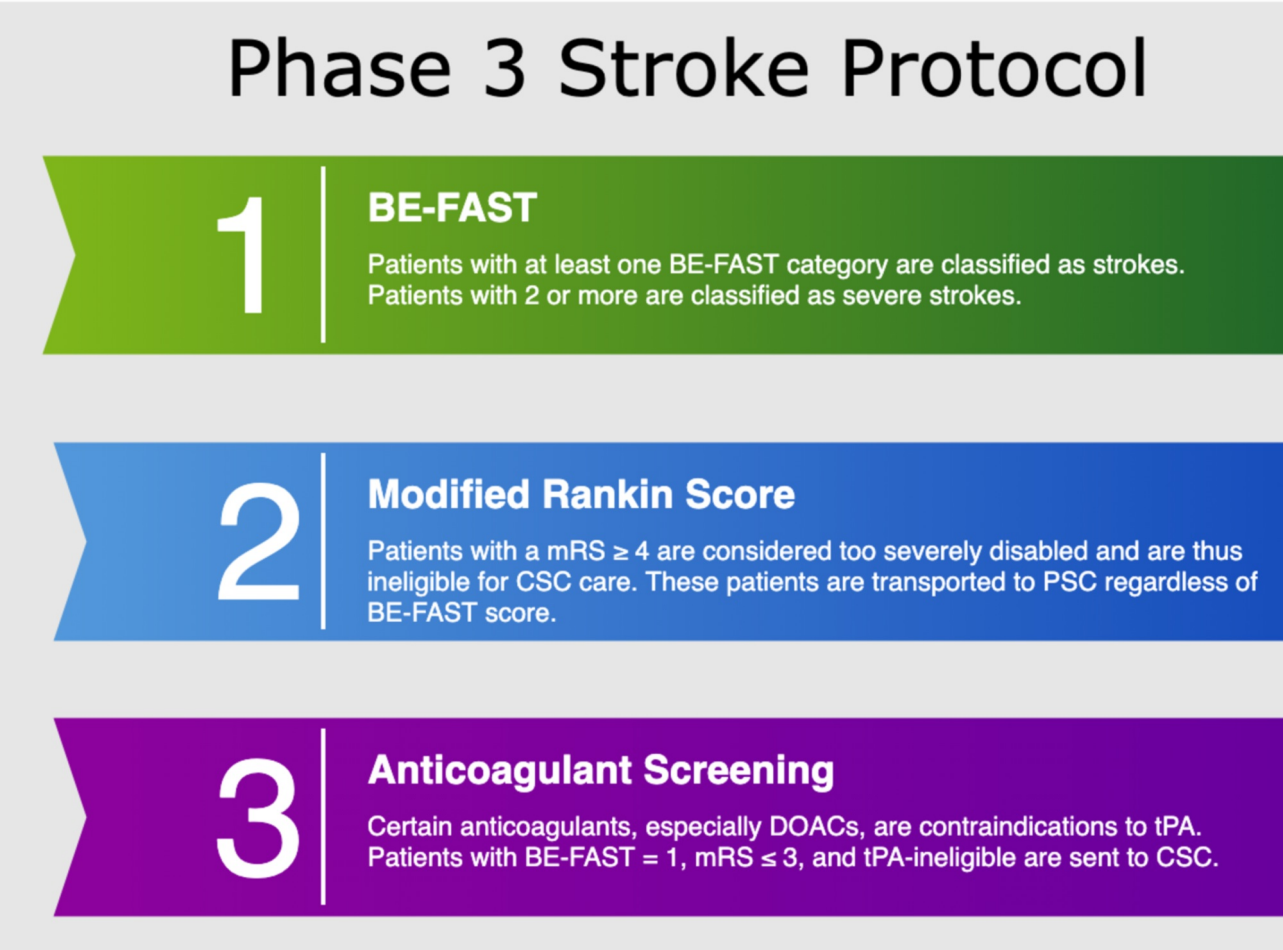
Phase 3 stroke protocol DOACs = direct oral anticoagulants, BE-FAST = balance, eyes, face, arm, speech, terrible headache/time to call 911, mRS = modified Rankin score, CSC = comprehensive stroke center, PSC = primary stroke center, tPA = tissue plasminogen activator

In order to reduce the risk of bias in our study, we made sure that every single patient transported for suspected stroke was enrolled and assessed with the prehospital scales. Before the data collection of Phase 1 began, every paramedic was given a one-hour hands-on training session on the stroke protocol, which included the LAMS, which was repeated annually. Prior to Phases 2 and 3, paramedics were also given one-hour training on the VAN and BE-FAST, respectively. No annual retraining was required for Phase 2, as it lasted only six months. Paramedics received annual retraining for the BE-FAST in Phase 3. Paramedics were not allowed to go on a stroke call until they had satisfied these training requirements. By enforcing annual hands-on training, and choosing simple and memorable scales, we aimed to minimize paramedic interuser variability. In addition to the stroke scales listed in the study, our stroke protocol employed the modified Rankin score (mRS) to exclude severely disabled patients from CSC care, and an anticoagulant checklist to send patients ineligible for tPA (but with a stroke score one point below the cutoff) to a CSC. Our stroke protocol in Phases 1 and 2 began with the BE-FAST, but only to classify patients as stroke alerts (Figure 2). In Phase 2, we used a combined scale with the LAMS and a modified VAN (Figure 3). Typically, the VAN is used as a test, however, in our protocol, we assigned one point to each of the three items. Additionally, the VAN items were analyzed and scored regardless of motor symptoms, as we used the LAMS to assess motor symptoms and sought to gather data in order to determine whether it is effective to combine the two scales, and if so, what the combination with the highest sensitivity and specificity is. In Phase 3, patients receive the BE-FAST, used as a scale from 0-6 (Figure 4).

PCFR has agreements with each of our local comprehensive and primary stroke receiving hospitals to provide outcome data, including NIHSS, at hospital arrival, any interventions performed, length of stay, ultimate diagnosis, and any discharge information. This allows us to correlate our stroke scale data with outcome data in order to evaluate the effectiveness of the three scales in our study.

## Results

**Figure 5:**
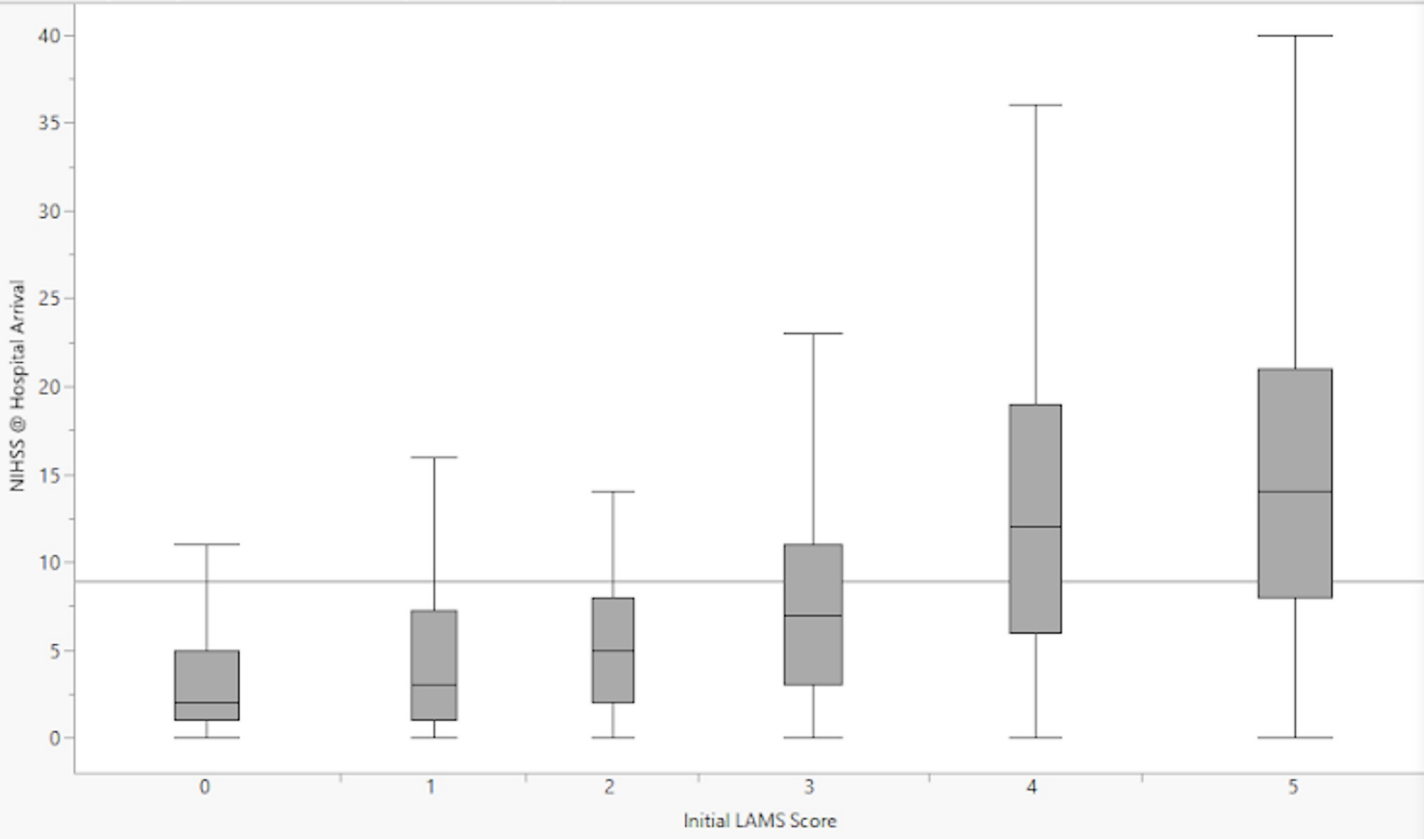
Box-and-whisker plots representing the distributions of NIHSS scores at hospital arrival for each prehospital LAMS score NIHSS = National Institutes of Health Stroke Scale; LAMS = Los Angeles motor scale

**Figure 6:**
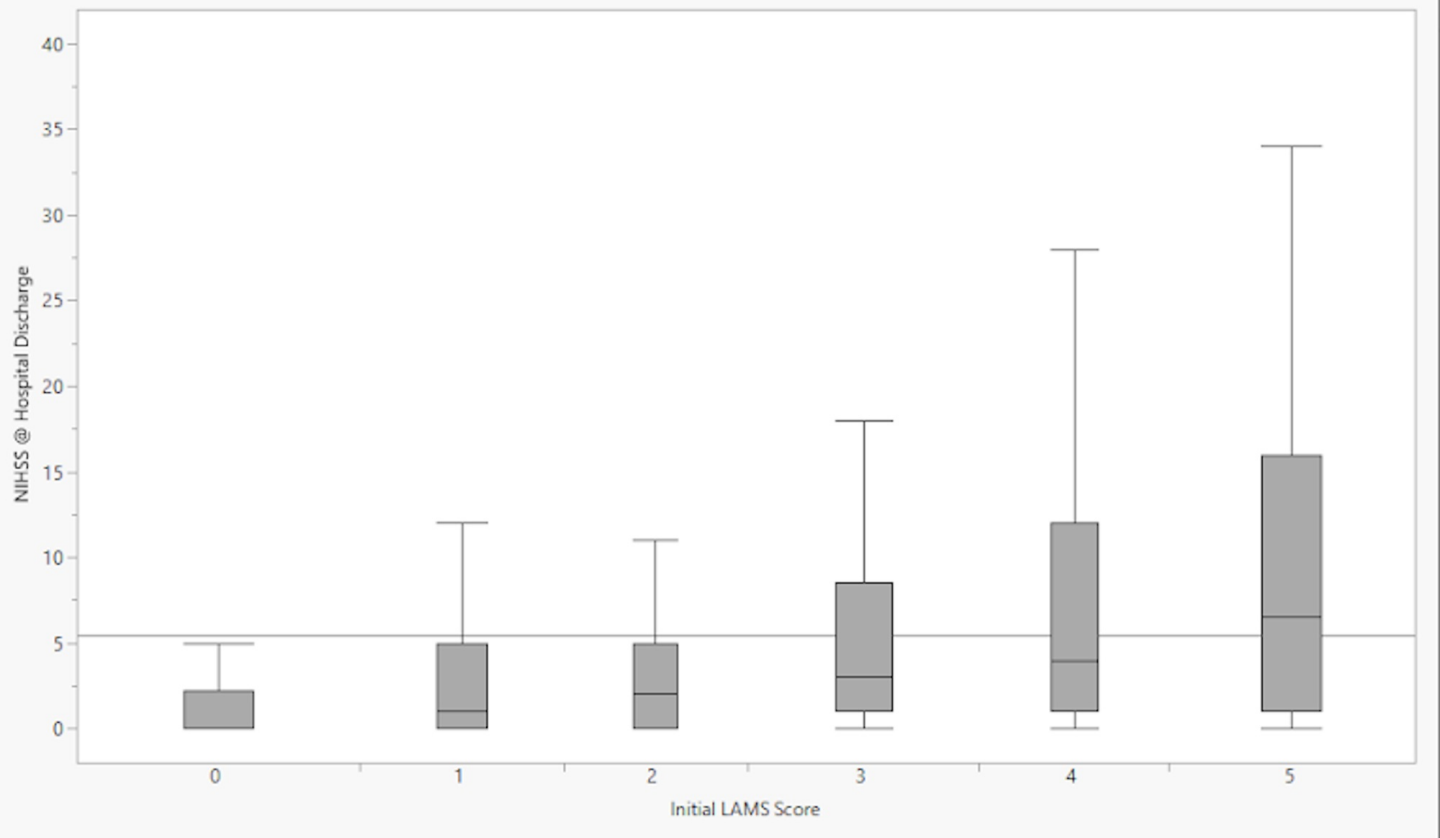
Box-and-whisker plots representing the distributions of NIHSS scores at hospital discharge for each prehospital LAMS score NIHSS = National Institutes of Health Stroke Scale; LAMS = Los Angeles motor scale

Phase 1 enrolled 2187 patients, Phase 2 enrolled 480 patients, and Phase 3 is ongoing. Out of the 2667 patients enrolled in Phases 1 and 2, 2374 (89%) had their LAMS score reported. Out of the 480 patients enrolled in Phase 2, 388 (81%) had their VAN score reported. Data for Phases 1 and 2 are undergoing analysis. We have included demographic data and baseline characteristics (Tables 1-2) for Phases 1 and 2, as well as box plots of LAMS score versus NIHSS at hospital arrival and hospital discharge (Figures 5-6).

**Table 1:** Demographics and baseline characteristics for all patients evaluated with LAMS (Phases 1 and 2) Numbers in parentheses in the "Category" field represent the number of patients for whom we had data in that category. The overall sample size was 2667 patients (the sum of Phases 1 and 2). IQR = interquartile range, CTA = computed tomography angiography, TIA = transient ischemic attack, SNF = skilled nursing facility, LAMS = Los Angeles motor scale, NIHSS = National Institutes of Health Stroke Scale

Category	Distribution
Sex (n = 2133)	49% male, 51% female
Age (n = 2665)	Median 72 years, IQR 60-81, range 13-108
LAMS score (n = 2374)	LAMS 0: 19%, LAMS 1: 14%, LAMS 2: 13%, LAMS 3: 17%, LAMS 4: 16%, LAMS 5: 21%
Received CTA perfusion imaging (n = 1706)	41% received, 59% did not
Received tPA (n = 2448)	12% received, 88% did not
Received mechanical intervention (n = 1780)	13% received, 87% did not
Stroke type diagnosis (n = 2414)	10% hemorrhagic, 45% ischemic, 10% TIA, 35% other/unknown
Discharge disposition (n = 2128)	49% discharged home, 19% to SNF, 13% to rehab, 7% expired, 12% to hospice or unknown
NIHSS at hospital arrival (n = 1537)	Median 6, IQR 2-13, range 0-40
NIHSS at hospital discharge (n = 1122)	Median 2, IQR 0-7, range 0-40

**Table 2:** Demographic and baseline characteristics for all patients who received both the LAMS and the VAN (Phase 2) Numbers in parentheses in the "Category" field represent the number of patients for whom we had data in that category. The overall sample size was 480 patients for Phase 2. IQR = interquartile range, CTA perfusion imaging = computed tomography angiography and perfusion imaging, TIA = transient ischemic attack, SNF = skilled nursing facility, NIHSS: National Institutes of Health Stroke Scale, LAMS = Los Angeles motor scale, VAN = vision, aphasia, neglect

Category	Distribution
Sex (n = 480)	50% male, 50% female
Age (n = 480)	Median 72, IQR 62-81, range 13-108
LAMS Score (n = 391)	LAMS 0: 22%, LAMS 1: 16%, LAMS 2: 14%, LAMS 3: 18%, LAMS 4: 14%, LAMS 5: 16%
Number of VAN categories satisfied (n = 388)	VAN 0: 42%, VAN 1: 38%, VAN 2: 18%, VAN 3: 2%
VAN + LAMS total score (n = 388)	Score 0: 12%, Score 1: 14%, Score 2: 17%, Score 3: 14%, Score 4: 15%, Score 5: 12%, Score 6: 11%, Score 7: 4%, Score 8: 1%
Received CTA perfusion imaging (n = 290)	26% received, 74% did not
Received tPA (n = 422)	14% received, 86% did not
Received mechanical intervention (n = 411)	7% received, 93% did not
Stroke type diagnosis (n = 423)	9% hemorrhagic, 43% ischemic, 11% TIA, 37% other/unknown
Discharge disposition (n = 422)	50% discharged home, 20% to SNF, 10% to rehab, 5% expired, 15% to hospice or unknown
NIHSS at hospital arrival (n = 398)	Median 6, IQR 2-13, range 0-36
NIHSS at hospital discharge (n = 321)	Median 2, IQR 0-7, range 0-40

## Discussion

A meta-analysis in 2018 identified LAMS, VAN, and NIHSS as the assessments with the highest sensitivity and specificity in predicting LVO [10]. However, the LAMS and NIHSS studies included were retrospective and do not appear to have been performed in the prehospital setting. The VAN studies, while prospective, had a total sample size of only 62 patients. Furthermore, this study did not include any trials on the BE-FAST. The large sample size of our study allows for a much smaller margin of error as compared to other prospective studies, which often enroll less than 100 patients. It is clear that there is overall insufficient research on prehospital stroke scales, and further studies must be performed to evaluate which one is most useful for use by EMS. 

A systematic review of the Cochrane database from 2019 noted that there is a lack of sufficient high-quality data to make a definitive conclusion on which prehospital stroke scale to use [6]. The studies included differ from our study for several reasons: 1) the systematic review does not consider stroke severity and is only focused on identifying strokes in the field; 2) none of the three scales we used were implemented (BE-FAST, LAMS, and VAN); 3) the sample sizes (range 31-1130, median 312) are significantly lower than ours; 4) a majority of the studies were classified as having high risk of bias. Note that the face, arm, speech, time (FAST) and the Los Angeles prehospital stroke Screen (LAPSS), which are related scales, were included in the study, but they are not equivalent to our scales (BE-FAST and LAMS). Additionally, both of these scales did not have high-quality data, with 4/5 LAPSS studies having a significant selection bias (as determined by the authors of the systematic review), and with the FAST data being inconsistent (sensitivity ranged from 0.13 to 0.92 across studies). To reduce the risk of selection bias, we have designed our protocol to enroll all suspected stroke patients as described in the following paragraph. In reality, 89% of patients in Phases 1 and 2 were evaluated with LAMS and 81% in Phase 2 were evaluated with VAN. This may be caused by paramedics not performing the stroke scales on every patient or by failing to record the results.

Our study is unique in that it is entirely conducted in the prehospital setting, and designed and implemented by our EMS agency. Given our direct relationship with the paramedics performing the stroke scales, we have significant involvement in the way they are performed, resulting in less variability. No paramedic was allowed to go on a stroke call until they had completed the one-hour annual hands-on training session. In order to ensure the quality of outcome data, every CSC and PSC we transport to is required to provide patient data to PCFR in order to continue to receive stroke transports from our EMS system. Every stroke transport was reviewed through a mandatory quality meeting within 45 days at the receiving hospital. Thus, we have taken measures to provide the most accurate data for our statistical analysis.

One area that this protocol does not encompass is the existence of thrombectomy-capable stroke centers (TSCs). These centers are capable of performing MT but cannot reliably treat hemorrhagic stroke patients. As these types of stroke centers (which result from PSCs becoming certified in thrombectomy) become more common, it is important to separately identify suspected hemorrhagic strokes so that they are not transported to a TSC, whereas LVO patients would receive adequate care at a TSC. Further research should be done to develop an effective protocol to utilize TSCs when patients are eligible while ensuring that non-LVO severe stroke patients can be taken to a CSC.

## Conclusions

The existing literature lacks sufficient high-quality data on prehospital stroke scales to determine which stroke scale should be performed by paramedics to determine transport decisions. Our prospective study, the LIT-PASS, is designed to alleviate the selection bias by including all suspected stroke patients. As the fourth largest EMS system in our state, we are able to achieve a high sample size and high-quality outcome data via agreements with receiving hospitals. When choosing stroke scales, simplicity should also be considered so that the paramedics can administer the scale quickly and reliably.
